# Identification of pathogenic germline variants in a large Chinese lung cancer cohort by clinical sequencing

**DOI:** 10.1002/1878-0261.13548

**Published:** 2024-01-25

**Authors:** Zhe Yu, Zirui Zhang, Jun Liu, Xiaoying Wu, Xiaojun Fan, Jiaohui Pang, Hua Bao, Jiani Yin, Xue Wu, Yang Shao, Zhengcheng Liu, Fang Liu

**Affiliations:** ^1^ Department of Respiratory Medicine Ningbo NO.2 Hospital China; ^2^ Department of Cardiovascular and Thoracic Surgery Nanjing Drum Tower Hospital Affiliated to Nanjing University School of Medicine China; ^3^ Department of Chemotherapy Affiliated Hospital of Nantong University China; ^4^ Nanjing Geneseeq Technology Inc. China; ^5^ School of Public Health Nanjing Medical University China; ^6^ Senior Department of Oncology The Fifth Medical Center of PLA General Hospital Beijing China

**Keywords:** DNA damage repair, germline variant, hereditary cancer syndrome, lung cancer

## Abstract

Genetic factors play significant roles in the tumorigenicity of lung cancer; however, there is lack of systematic and large‐scale characterization of pathogenic germline variants for lung cancer. In this study, germline variants in 146 preselected cancer‐susceptibility genes were detected in 17 904 Chinese lung cancer patients by clinical next‐generation sequencing. Among 17 904 patients, 1738 patients (9.7%) carried 1840 pathogenic/likely pathogenic (P/LP) variants from 87 cancer‐susceptibility genes. *SBDS* (SBDS ribosome maturation factor) (1.37%), *TSHR* (thyroid stimulating hormone receptor) (1.20%), *BLM* (BLM RecQ like helicase) (0.62%), *BRCA2* (BRCA2 DNA repair associated) (0.62%), and *ATM* (ATM serine/threonine kinase) (0.45%) were the top five genes with the highest overall prevalence. The top mutated pathways were all involved in DNA damage repair (DDR). Case–control analysis showed *SBDS* c.184A>T(p.K62*), *TSHR* c.1574T>C(p.F525S), *BRIP1* (BRCA1 interacting helicase 1) c.1018C>T(p.L340F), and *MUTYH* (mutY DNA glycosylase) c.55C>T(p.R19*) were significantly associated with increased lung cancer risk (*q* value < 0.05). P/LP variants in certain genes were associated with early onset of lung cancer. Our study indicates that Chinese lung cancer patients have a higher prevalence of P/LP variants than previously reported. P/LP variants are distributed in multiple pathways and dominated by DNA damage repair‐associated pathways. The association between identified P/LP variants and lung cancer risk requires further studies for verification.

## Graphical abstract

This research aimed to identify susceptibility germline variants in a large cohort of Chinese lung cancer patients. Pathogenic and likely pathogenic variants were notably prevalent in DNA damage repair pathways. Through case–control analysis, we identified certain variants that association with an elevated risk of lung cancer. Furthermore, our investigation revealed that some germline variants may affect the somatic mutation spectrum of certain genes and somatic mutation signatures.

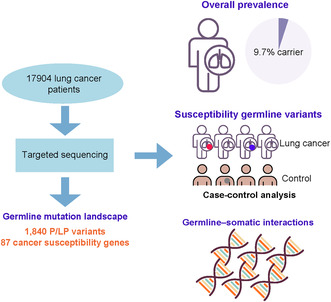

**Abbreviations**
AD: allele depth
AF: allele frequency
APOBEC: apolipoprotein B mRNA‐editing enzyme catalytic polypeptide
ARID1A: AT‐rich interaction domain 1A
ATM: ATM serine/threonine kinase
BLM: BLM RecQ like helicase
BRCA1: BRCA1 DNA repair associated
BRCA2: BRCA2 DNA repair associated
BRIP1: BRCA1 interacting helicase 1
CAP: College of American Pathologists
CDC73: cell division cycle 73
CDS: coding sequence
CHEK2: checkpoint kinase 2
CIS: chromosomal instability score
CLIA: Clinical Laboratory Improvement Amendments
CNVs: copy number variations
CTCF: CCCTC‐binding factor
DDR: DNA damage repair
EGFR: epidermal growth factor receptor
ERBB2: erb‐b2 receptor tyrosine kinase 2
ERCC3: ERCC excision repair 3, TFIIH core complex helicase subunit
ERCC5: ERCC excision repair 5, endonuclease
EZH2: enhancer of zeste 2 polycomb repressive complex 2 subunit
FA: Fanconi anemia pathway
FANCA: FA complementation group A
FANCD2: FA complementation group D2
FANCL: FA complementation group L
FAT1: FAT atypical cadherin 1
GWAS: genome‐wide association study
HR: homologous recombination repair
indels: insertions/deletions
JAK2: Janus kinase 2
LASC: lung adenosquamous carcinoma
LB: likely benign
LP: likely pathogenic
LUAD: lung adenocarcinoma
LUSC: lung squamous cell carcinoma
MLH3: mutL homolog 3
MMR: mismatch repair
MPCs: multiple primary cancers
MUTYH: mutY DNA glycosylase
NER: nucleotide excision repair
NGS: next generation sequencing
P/LP: pathogenic/likely pathogenic
PALB2: partner and localizer of BRCA2
PCR: polymerase chain reaction
POLE: DNA polymerase epsilon, catalytic subunit
POLH: DNA polymerase eta
PRF1: perforin 1
SBDS: SBDS ribosome maturation factor
SBS: single base substitution
SCLC: small cell lung cancer
SMARCA4: SWI/SNF related, matrix associated, actin dependent regulator of chromatin, subfamily a, member 4
SNPs: single nucleotide polymorphisms
SNVs: single nucleotide variants
TGFBR2: transforming growth factor beta receptor 2
TMB: tumor mutation burden
TP53: tumor protein p53
TSHR: thyroid stimulating hormone receptor
VUS: variant of unknown significance
WBC: white blood cell
WRN: WRN RecQ like helicase
XPA: XPA, DNA damage recognition and repair factor
XPC: XPC complex subunit, DNA damage recognition and repair factor

## 1 Introduction

It is well known that environmental factors such as smoking, ionizing radiation, occupational exposure, and air pollution are the primary risk factors for the development of lung cancer [1]. However, more and more evidence supports the significant role of genetic factors in the tumorigenicity of lung cancer [2]. Early studies found that the risk of lung cancer increased 1.51‐fold in people with a first‐degree relative with lung cancer after controlling smoking and other factors [3], and lung cancer also had a higher heritability (18%) compared with colorectal cancer (14–15%) revealed by a twin study [4], both of which clearly show the genetic susceptibility of lung cancer. Currently, there are two ways to be used in the identification of genetic risk factors of cancer. The first is genome‐wide association study (GWAS), which is applied to explore the genetic predisposition of common genetic variants for cancer. Up to now, over 50 susceptibility single nucleotide polymorphisms (SNPs) have been identified to be associated with lung cancer risk in Chinese and European populations [5, 6, 7, 8, 9]. The second is to identify pathogenic/likely pathogenic (P/LP) germline variants in cancer susceptibility genes, making it possible to explore the functional impact of rare and very rare germline variants in the cancer development [10, 11]. For lung cancer, *EGFR* T790M germline mutation has a prevalence of 0.3–0.9% in lung adenocarcinoma (LUAD) [2, 11, 12], and the germline T790M mutation is associated with female gender, multifocal lesions, and never smoker [13]. In addition of T790M, other germline variations in *EGFR* have been reported, for example, R776H, R776G, V843I, and V834L. [14]. Pathogenic germline variations in other cancer susceptibility genes are also found to be associated with lung cancer risk including *TP53* (i.e., Li‐Fraumeni syndrome), *ATM* [15, 16], *BRAC2*, and *CHEK2* [17, 18].

Race/ethnicity‐associated differential genetic background affects predisposition variant identification. For instance, germline mutations in *BRAC2* were found to be associated with a higher risk and early onset of lung cancer in Chinese population [19, 20]; however, such an association was not observed in several studies with European ancestry as study subjects [2], emphasizing the need to characterize the landscape of ethnicity‐specific harmful inherited genetic variations. Some studies have been conducted to pinpoint genes and loci associated with susceptibility to lung cancer in Chinese population [9, 20, 21, 22, 23, 24]. Existing studies have some limitations such as small sample size, few covered genes, and lack of somatic mutation data, reducing the ability of finding rare germline variants and the exploration of the interaction between germline and somatic mutations.

In this study, we retrospectively reviewed germline mutations in 146 canonical cancer susceptibility genes in 17 904 Chinese lung cancer patients, analyzed the prevalence of the P/LP germline mutations, assessed their risk for lung cancer by comparing to the gnomAD East Asian population cohort and investigated the association between germline and somatic mutations in this population.

## 2 Materials and methods

### 2.1 Cohort information

**Fig. 1:**
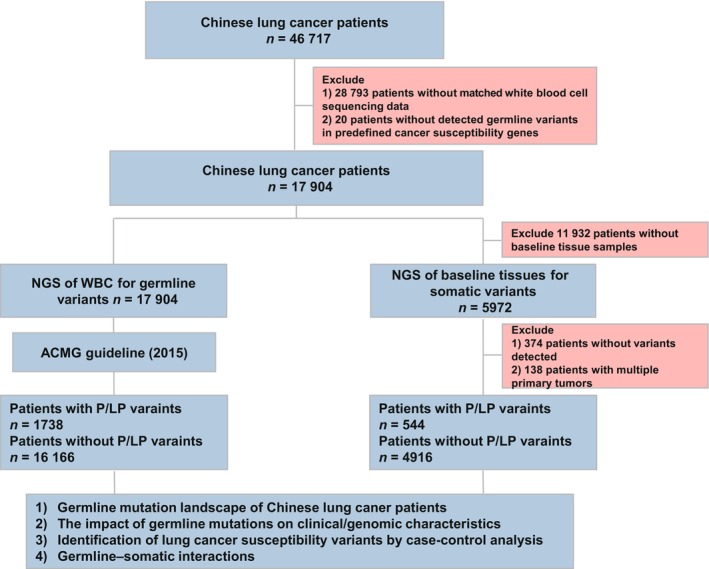
Study diagram and patient selection. 46 717 lung cancer patients undergoing targeted sequencing as part of clinical care from May 2016 to April 2022 were screened, and a total of 17 904 patients were included in the germline variant analysis. The pathogenicity of the germline variants was annotated with ACMG guidelines. Moreover, there were 5460 patients who were included in the germline‐somatic interaction analysis. ACMG, American College of Medical Genetics and Genomics.

We first screened 46 717 lung cancer patients from multiple institutions, including Nanjing Drum Tower Hospital Affiliated to Nanjing University School of Medicine, The Fifth Medical Center of PLA General Hospital, Ningbo NO.2 Hospital, Affiliated Hospital of Nantong University et al., who underwent deep targeted sequencing of tissue samples for treatment purpose between May 2016 to April 2022. There were 28 793 patients who had no matched white blood cell sequencing data and these patients were excluded, leaving 17 924 patients for the next‐step screening. Among 17 924 patients, 1758 patients were detected with germline P/LP mutations; however, the mutations in 20 patients were not in the predefined cancer susceptibility genes and these 20 patients were excluded. In the end, a total of 17 904 patients were included in the study, 1738 of whom were detected with germline P/LP mutations in at least one of the 146 cancer susceptibility genes, and 16 166 of whom were not detected with germline P/LP mutations. For germline/somatic interaction analysis, among 17 904 patients, 5972 patients also had baseline (pretreatment) tissue sequencing data, and out of whom, 374 patients without detectable somatic mutations in the tissue samples were excluded for the analysis. In addition, for 138 patients with multiple primary cancers (MPCs), since the somatic mutation information of these patients was incomplete (they usually only had one cancer sample to be sequenced in the laboratory and the somatic mutations in other primary cancers were unknown), they were also excluded; at last, 5460 patients with detectable somatic mutations in the baseline tissue samples were included in the germline/somatic interaction analysis. Patient screening procedures and study design were shown in Fig. 1. The study methodologies conformed to the standards set by the Declaration of Helsinki. All patients signed informed consent for research‐purpose use of their de‐identified sequencing data. The study was categorized as a noninterventional retrospective study and did not involve the use of sensitive personal data or any commercial interests. The study was approved by the Medical Ethics Committee of Nanjing Geneseeq Medical Laboratory, which was responsible for clinical sequencing, generation and governance of sequencing data in the study (Approval number: NSJB‐MEC‐2023‐06).

### 2.2 Targeted sequencing and variant calling

Library construction and targeted sequencing were performed according to the method described previously [25]. Briefly, genomic DNAs from tissue samples and matched white blood cell (WBC) samples were extracted by using of dsDNA HS Assay Kit (Thermo Fisher Scientific, Waltham, MA, USA) and quantified by Qubit V.3.0. Library was prepared by using of KAPA Hyper Prep Kit (KAPA Biosystems, Wilmington, MA, USA). Customized xGen lockdown probes (Integrated DNA Technologies, Coralville, IA, USA) that covered all exons and selected introns of 425, 416, 422 or 474 cancer‐related genes were used for hybridization capture enrichment (As the sequencing panels are expanded and updated with time, multiple targeted panels were used during the study period). Next‐generation sequencing (NGS) tests were performed at a Clinical Laboratory Improvement Amendments (CLIA) licensed, ISO 15189:2012, and College of American Pathologists (CAP)‐accredited laboratory (Nanjing Geneseeq Medical Laboratory, China). Paired‐end sequencing was performed on the Illumina HiSeq4000 platform (Illumina, San Diego, CA, USA) after PCR amplification and purification. burrows‐wheeler aligner [26] was used to align paired‐end sequencing reads to the reference human genome (hg19), and duplication mark was performed by sambamba [27]. genome analysis toolkit (gatk 3.4.0) [28] was used for local realignment around insertions/deletions and base quality score recalibration. For germline variants, varscan2 [29] was used to identify single nucleotide variants (SNVs) and small insertions/deletions (indels) from the WBC DNA samples with the cut‐off value of allele depth (AD) ≥ 8 and allele frequency (AF) ≥ 15%. varscan2 was also used to call somatic SNVs and indels, and somatic gene fusion variants were called by factera [30] with default parameters. Germline and somatic variants were annotated by Annovar [31], ClinVar [32], HGMD [33], LOVD [34], 1000g [35], ExAC [36], and gnomAD [37]. Variants with ≥ 1% frequency in any of the following four databases including in‐house database, 1000g, ExAC and gnomAD were removed. In addition, the functional impact of the variants was annotated by PolyPhen [38], SIFT [39], CADD [40], and MutationTaster [41]. Somatic copy number variations (CNVs) were called by cnvkit [42] with default parameters and chromosomal instability score (CIS) was defined as the proportion of abnormal segments (log_2_ depth ratio > 0.2 or < −0.2) accounting for 22 autosomes. Tumor mutation burden (TMB) was calculated according to the method previously described [43].

### 2.3 Gene annotation and germline P/LP variant annotation

Cancer susceptibility genes were selected based on the NCCN Guidelines for Genetics/Familial High‐Risk Assessment (https://www.nccn.org/guidelines/category_2) and previous literatures [10, 44]. The cancer susceptibility gene list contains 215 genes. Considering that multiple targeted sequencing gene panels were used in this cohort, we only investigated the intersection of the multiple gene panels and the cancer susceptibility gene list; as a result, the intersection contained a total of 146 genes (Table S1). Among the 146 genes, germline P/LP variants were detected in 87 genes; therefore, the 87 genes were chosen for further annotation and analysis. The 87 genes were mapped to the 10 predefined oncogenic signaling pathways according to the literature [45]. In addition, as the 10 oncogenic pathways do not include DNA damage repair, we further annotated cancer susceptibility genes involved in DNA damage repair to damage sensor, homologous recombination repair (HR), mismatch repair (MMR), Fanconi anemia pathway (FA), nucleotide excision repair (NER), and other DDRs according to the literature [46]. For damage sensor pathway, only two genes, *ATM* and *CHEK2*, were included. The two genes also belong to the TP53 pathway, that is, damage sensor pathway is a subset of the TP53 pathway. The genes were annotated as oncogene or tumor suppressor gene by reference to previous literature [10, 47]. Cancer SIGVAR [48], a semiautomated annotation tool for the pathogenicity of germline variants which implements the framework of the ACMG/AMP guidelines [49], was used to annotate the pathogenic status of the germline variants. The variants were assigned five classes including benign (B), likely benign (LB), variant of unknown significance (VUS), likely pathogenic (LP), and pathogenic (P).

### 2.4 Mutation signature analysis

Given the targeted sequencing in this study, the low mutation numbers in each sample did not allow us to do reliable mutational signature analysis for individual samples. We thereby combined SNVs from all samples with or without germline variants in certain pathways (for example, samples with germline mutations in the FA pathway *vs*. samples without germline mutations in the FA pathway) as described in the study of Peng et al. [20]. sigminer package [50] was used to extract 30 COSMIC single base substitution (SBS) signatures from the pooled SNVs. The contributions of the signatures with same etiological origin were combined, for example, SBS6, 15, 20, and 26 were extracted and they are all associated with MMR deficiency, therefore, the contributions of the four signatures were summed and represented the contribution of MMR deficiency signature. Only pathways with germline mutations in at least five samples and > 100 pooled germline SNVs were included.

### 2.5 Mutually exclusive and co‐occurrence of germline variants and somatic variants

Mutually exclusive and co‐occurrence of germline variants and somatic variants were investigated using rediscover package [51]. We conducted germline gene/somatic gene pair and germline pathway/somatic gene pair analysis. The function “getMutexAB” was used to extract significant mutually exclusive or co‐occurrent germline gene (or pathway)/somatic gene pairs based on the two matrices model.

### 2.6 Statistical analysis

The association between germline P/LP variants and lung cancer risk was evaluated by a case–control analysis with the East Asian population dataset of the Genome Aggregation Database (gnomAD) as the control cohort. To compare the prevalence of the identified germline P/LP variants in the case cohort (the local lung cancer cohort) and the control cohort, we referred to the one‐tailed Fisher's exact test used in previous studies [10, 52]. The alternative hypothesis is that a tested germline P/LP variant has a higher prevalence in the case cohort than the control cohort, that is, the variant is enriched in the case cohort. For other statistical tests, chi‐squared test was used for the intergroup comparison of the proportions of categorical variables (Fisher's exact test was used if one or more expected cell count was < 5) and Wilcoxon rank sum test was used for the intergroup comparison of continuous variables. Kolmogorov–Smirnov test was used for the comparison of the mutation position distribution of a gene in two groups (for example, with and without a certain P/LP germline variant). Two‐tailed test was used unless otherwise specified. Benjamini–Hochberg method was used for multiple test adjustment to control the false discovery rate. All statistical analyses were performed in r 4.2.0.

## 3 Results

### 3.1 Cohort information

After filtering (refer to “Materials and methods”), a total of 17 904 Chinese lung cancer patients were included in the study. There were 711 patients (4.0%) with early‐onset lung cancer, that is, < 40 years old at diagnosis, and 14 683 (82%) patients were ≥40 years old at diagnosis. The median age was 61 years old. Male patients accounted for 58.1% (*n* = 10 404) and female patients accounted for 41.9% (*n* = 7494). As to histological type, majority were LUAD (65.6%, *n* = 11 740), followed by lung squamous cell carcinoma (LUSC) (8.7%, *n* = 1557), small cell lung cancer (SCLC) (2.3%, *n* = 405), and lung adenosquamous carcinoma (LASC) (1.1%, *n* = 188). Stage I, II, III, and IV accounted for 2.5% (*n* = 448), 1.2% (*n* = 221), 3.8% (*n* = 672) and 26.6% (*n* = 4771) respectively. There were 4929 (27.5%) patients with a family history of cancer, and among them, 2052 (11.5%) patients had a family history of lung cancer. A few of patients (1.9%, *n* = 341) had MPCs. Cohort information was shown in Table 1.

**Table 1:** Clinical characteristics of the included patients.

	All (*N* = 17 904)
Age at diagnosis	
< 40	711 (4.0%)
≥ 40	14 683 (82%)
Unknown	2510 (14.0%)
Median (range, years)	61 (14–97)
Sex	
Female	7494 (41.9%)
Male	10 404 (58.1%)
Unknown	6 (0.0%)
Stage	
I	448 (2.5%)
II	221 (1.2%)
III	672 (3.8%)
IV	4771 (26.6%)
Unknown	11 792 (65.9%)
Histological subtype	
LUAD	11 740 (65.6%)
LUSC	1557 (8.7%)
LASC	188 (1.1%)
SCLC	405 (2.3%)
Others	197 (1.1%)
Unknown	3817 (21.3%)
Number of cancers	
Single cancer	17 563 (98.1%)
Multiple primary cancers	341 (1.9%)
Family cancer history	
Yes	4929 (27.5%)
No	8054 (45.0%)
Unknown	4921 (27.5%)
Family lung cancer history	
Yes	2052 (11.5%)
No	2877 (16.1%)

### 3.2 Prevalence of germline P/LP variants in Chinese lung cancer patients

**Fig. 2:**
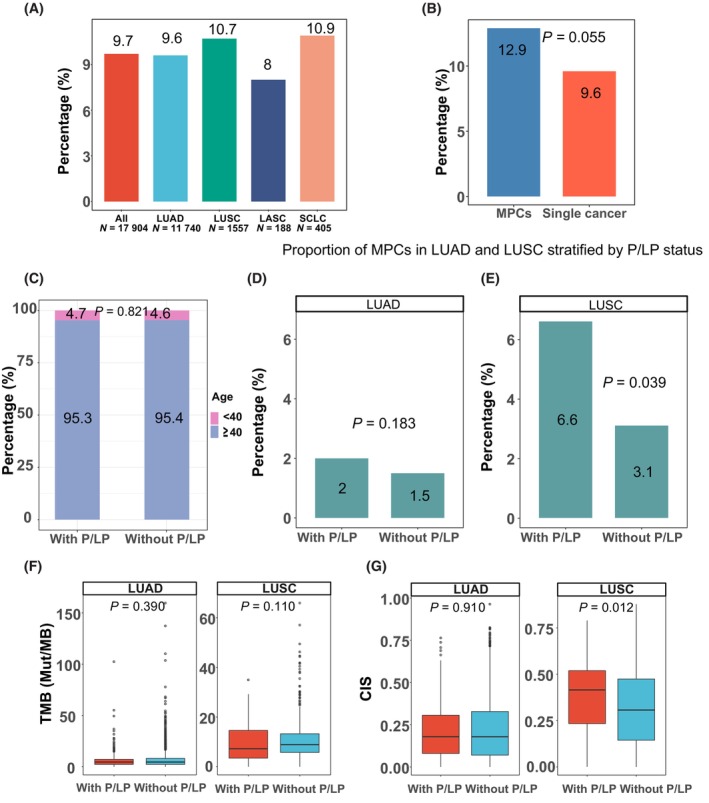
Prevalence of germline P/LP variants in Chinese lung cancer patients. (A) The prevalence of P/LP variants in general and their prevalence across different histological subtypes (Chi‐squared test). (B) The prevalence of P/LP variants in the patients with MPCs and with single primary cancer (Chi‐squared test). (C) Age distribution in the patients with P/LP variants and without P/LP variants (Chi‐squared test). (D) Proportion of MPCs in LUAD stratified by P/LP status (Chi‐squared test). (E) Proportion of MPCs in LUSC stratified by P/LP status (Fisher's test). (F) TMB in the patients with and without P/LP variants in the LUAD and LUSC group (Wilcoxon rank sum test). (G) CIS in the patients with and without P/LP variants in the LUAD and LUSC group (Wilcoxon rank sum test). ALL, all lung cancer patients.

Among 17 904 patients, 1738 patients (9.7%) carried 1840 P/LP variants from 87 cancer susceptibility genes, and the prevalence of germline P/LP mutations in the LUAD, LUSC, LASC, and SCLC patients were 9.6%, 10.7%, 8%, and 10.9% respectively, without showing significant difference (*P* = 0.77) (Fig. 2A). In addition, the patients with MPCs had a higher detection rate of germline P/LP variants (12.9%) compared to those with single cancer (9.6%) (*P* = 0.055) (Fig. 2B). Detailed information of the 87 cancer susceptibility genes were provided in Table S2. The proportions of early‐onset (< 40) and late‐onset (≥ 40) patients were similar in the P/LP group and non‐P/LP group and did not show significant difference either (Fig. 2C). The P/LP group had a higher proportion of the patients with MPCs compared with the non‐P/LP group (2% *vs*. 1.5% in LUAD and 6.6% *vs*. 3.1% in LUSC) and the difference was significant in LUSC (*P* = 0.039) (Fig. 2D,E). Furthermore, while there was no association between TMB or CIS and the status of P/LP mutations in LUAD, such association appeared to exist in LUSC. The LUSC patients with P/LP mutations had a lower TMB with a borderline *P* value (*P* = 0.110) and a significant higher CIS (*P* = 0.012) than the LUSC patients without P/LP mutations (7 *vs*. 9 for TMB and 0.42 *vs*. 0.31 for CIS, for the LUSC patients with and without P/LP mutations respectively) (Fig. 2F,G). The status of P/LP mutations did not show significant association with other clinical features.

### 3.3 Landscape of germline P/LP variants in Chinese lung cancer patients

**Fig. 3:**
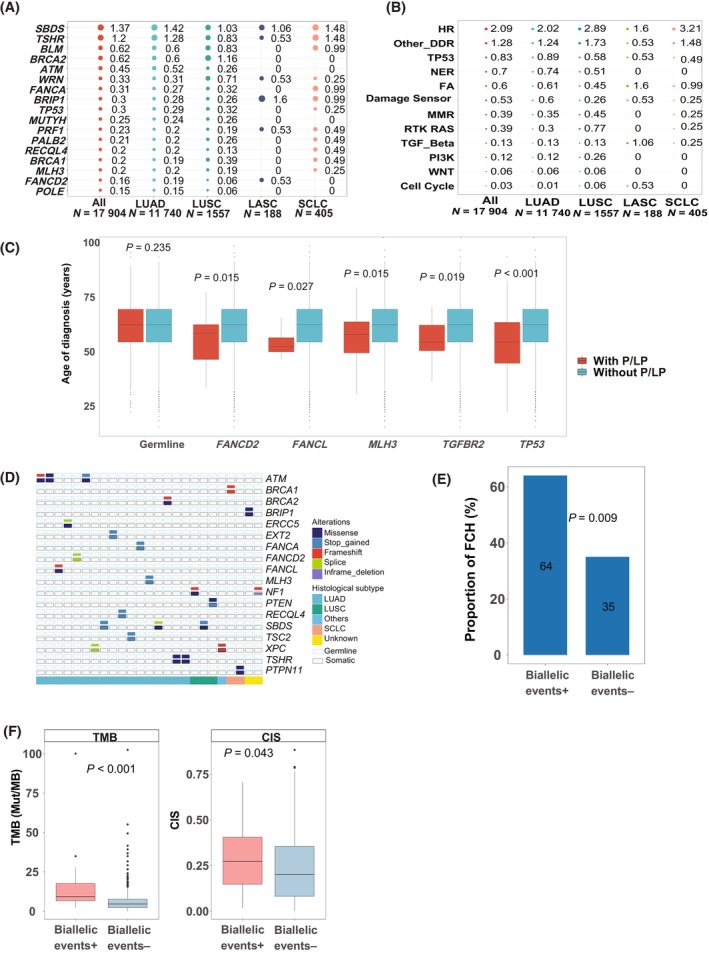
Landscape of germline P/LP variants and biallelic events. (A) Genes that have germline P/LP variants with an overall prevalence ≥ 0.15% and their prevalence in various histological subtypes (Fisher's test). (B) The prevalence of P/LP variants in different pathways. (C) Genes of which germline P/LP variants are linked to the earlier onset of lung cancer (Wilcoxon rank sum test). *P* values are not adjusted. (D) Oncoprint plot shows the genes and patients with biallelic events. (E) Proportion of the patients with a FCH in the biallelic event positive group and biallelic event negative group (Fisher's test). (F) Comparison of TMB and CIS between the biallelic event positive group and biallelic event negative group (Wilcoxon rank sum test). ALL, all lung cancer patients; Cell Cycle, Cell Cycle pathway; Damage Sensor, Damage Sensor pathway; FCH, family cancer history; other_DDR, other DNA damage repair pathways; PI3K, PI3K pathway; RTK RAS, RTK RAS pathway; TGF_Beta, TGF‐Beta pathway; TP53, TP53 pathway; WNT, WNT pathway.

We identified cancer susceptibility genes with a high frequency of germline P/LP mutation in this cohort. Seventeen genes had a ≥ 0.15% overall mutation frequency and were listed in Fig. 3A. *SBDS* gene had the highest mutation frequency (1.37%) in the whole cohort as well as LUAD (1.42%) and SCLC (1.48%) subtype among all included genes. Other frequently mutated genes also included *TSHR*, *BLM*, *BRCA2*, *ATM*, *WRN*, *FANCA*, etc. (Fig. 3A). Some genes showed variation of mutation frequency in different histological subtypes. For example, *BRCA2* had a mutation frequency of 0.6% in LUAD and 1.16% in LUSC (*P* = 0.02, although not significant after multiple test adjustment). *BRCA2* was also the gene with the highest mutation frequency in LUSC (Fig. 3A). *BRIP1* had a relatively high mutation frequency in LASC (1.6%) and SCLC (0.99%) compared with LUAD (0.28%) and LUSC (0.26%). The mutation frequencies of all 87 genes in the four histological subtypes were shown in the Table S2. At pathway level, all of the seven pathways involved in DNA damage repair (DDR) ranked the most frequently mutated pathways (Fig. 3B). HR pathway had the highest overall mutation frequency as well as in all histological subtypes. LASC and SCLC exhibited distinct germline P/LP variant landscape in comparison with LUAD and LUSC. As to DDR pathways, except HR which showed the highest mutation frequency in all subtypes, the mutation frequency of MMR and NER pathways was higher in both LUAD (0.35% and 0.74%) and LUSC (0.45% and 0.51%) compared to LASC (0% and 0%) and SCLC (0.25% and 0%), whereas the mutation frequency of FA was higher in LASC (1.6%) and SCLC (0.99%) than in LUAD (0.61%) and LUSC (0.45%). In addition, cell cycle showed a high mutation frequency in LASC (0.53%) in comparison with other subtypes (0.01%, 0.06% and 0% for LUAD, LUSC and SCLC) (Fig. 3B), suggesting a heterogeneity of germline P/LP mutation profile in various histological subtypes.

Although the overall status of germline P/LP mutation was not associated with the age at diagnosis, P/LP mutations in certain genes was correlated with a younger age at diagnosis including *FANCD2*, *FANCL*, *MLH3*, *TGFBR2*, and *TP53* (Fig. 3C). We also investigated biallelic events which were defined as the presence of both germline and somatic mutations in same gene in a patient. For the patients with single primary cancer, among 542 evaluable patients (those with both germline and somatic mutations which were in same or different genes), 25 patients (4.6%) had biallelic events, and the involved genes included *ATM*, *BRCA1*, *BRCA2*, *BRIP1*, and *ERCC5*. (Fig. 3D). Although *EGFR* germline mutations have been reported in previous literature, there was only one case of *EGFR* germline P/LP mutation in our cohort; therefore, we did not find biallelic events in *EGFR* gene. Further analysis showed that patients with biallelic events were more likely to have a family history of cancer (not limited in lung cancer) (Fig. 3E). 64% of the patients with biallelic events had a family history of cancer in contrast with only 35% of the patients without biallelic events (*P* = 0.009). Biallelic events were also correlated with the higher TMB and CIS (Fig. 3F), probably because tumors with high genomic instability have a higher chance to generate the second mutation. For 341 patients with MPCs, 138 had available somatic mutation data. 20 of the 138 patients had both P/LP germline and somatic mutations, and biallelic event was found in only one patient (*BRCA1* gene). The 5% (1/20) percentage is not higher than the 4.6% percentage (25/542) in the patients with single primary cancer (lung cancer). It should be noted that for the patients with MPCs, their somatic mutation information was incomplete (generally, mutation information of only one primary cancer was available), biallelic event rate in these patients may be underestimated.

We evaluated the association between germline P/LP variants and lung cancer risk by a case–control analysis in which the gnomAD East Asian dataset acted as the control cohort. After multiple test adjustment, there were nine variants with < 0.25 *q* value, and four variants with < 0.05 *q* value among them (Table 2). The four variants included SBDS c.184A>T(p.K62*), TSHR c.1574T>C(p.F525S), MUTYH c.55C>T(p.R19*), and BRIP1 c.1018C>T(p.L340F), and the corresponding odds ratios were 4.8, 2.07, 9.76, and 4.63 respectively. The other five variants included JAK2 c.1849G>T(p.V617F), PRF1 c.305G>T(p.C102F), BLM c.2371C>T(p.R791C), TGFBR2 c.95‐2A>G and ERBB2 c.1397C>T(p.A466V), and the odds ratios ranged 1.61–4.11. SBDS p.K62* truncation variant is more common in East Asia population (0.033% of AF) than European population (0.015%), and in our lung cancer cohort, the AF of the variant was as high as 0.156%. Other variants except JAK2 c.1849G>T(p.V617F) which has a higher prevalence in European population than East Asia population, showed the same trend.

**Table 2:** Germline P/LP variants associated with lung cancer risk. AC, allele count; AN, allele number; CI, confidence interval; eas, East Asian; nfe, European (non‐Finnish); OR, odds ratio.

Germline P/LP variant	Local dataset (case group)			gnomAD east Asian dataset (control group)			Case–control analysis			gnomAD European (non‐Finnish) dataset		
	AC	AN	AF	AC eas	AN eas	AF eas	Fisher test (greater)			AC nfe	AN nfe	AF nfe
							*P* value	OR (95% CI)	FDR			
SBDS c.184A>T(p.K62*)	56	35 808	0.001564	6	18 378	0.000326	9.7E‐06	4.8 (2.31–Inf)	0.001	17	113 684	0.00015
TSHR c.1574T>C(p.F525S)	133	35 808	0.003714	33	18 382	0.001795	4.8E‐05	2.07 (1.49–Inf)	0.001	0	113 702	0
MUTYH c.55C>T(p.R19*)	19	35 808	0.000531	1	18 394	5.44E‐05	0.003	9.76 (1.86–Inf)	0.04	0	113 754	0
BRIP1 c.1018C>T(p.L340F)	27	35 808	0.000754	3	18 394	0.000163	0.003	4.63 (1.64–Inf)	0.04	0	113 628	0
JAK2 c.1849G>T(p.V617F)	21	35 808	0.000586	3	18 368	0.000163	0.017	3.59 (1.24–Inf)	0.194	45	113 248	0.000397
PRF1 c.305G>T(p.C102F)	9	35 808	0.000251	0	18 302	0	0.024	Inf (1.29–Inf)	0.195	0	112 238	0
BLM c.2371C>T(p.R791C)	75	35 808	0.002095	24	18 394	0.001305	0.024	1.61 (1.07–Inf)	0.195	2	113 748	1.76E‐05
TGFBR2 c.95‐2A>G	16	35 808	0.000447	2	18 388	0.000109	0.029	4.11 (1.14–Inf)	0.204	0	113 258	0
ERBB2 c.1397C>T(p.A466V)	15	35 808	0.000419	2	18 392	0.000109	0.04	3.85 (1.06–Inf)	0.247	0	113 648	0

### 3.4 Germline variants impact on somatic mutation profile

**Fig. 4:**
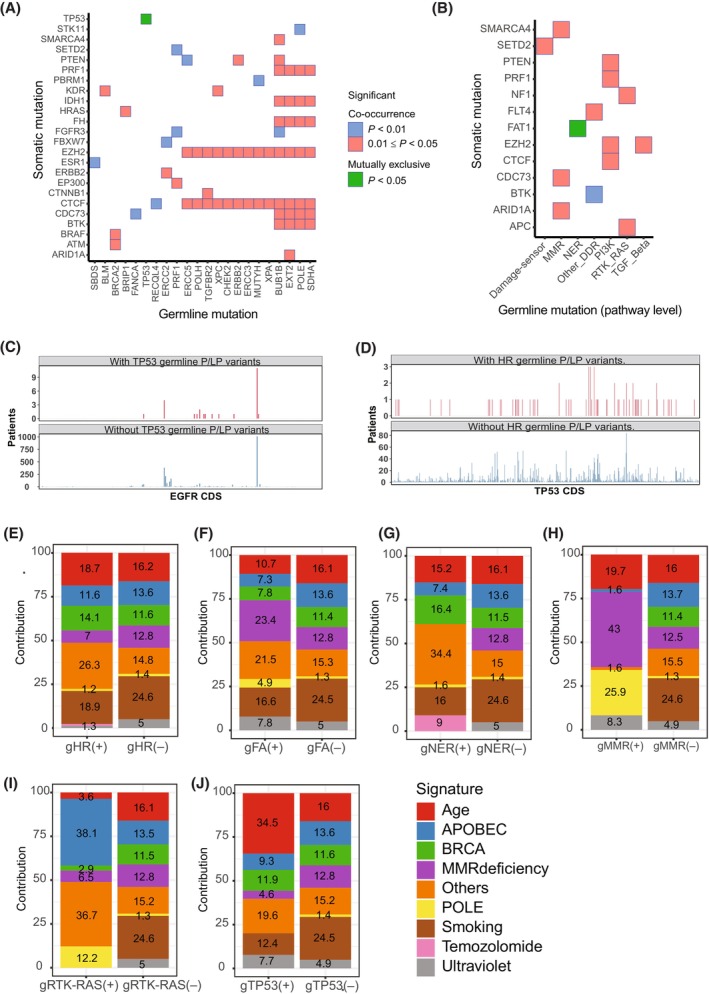
Impact of germline variants on somatic mutability. (A) Co‐occurrent and mutually exclusive association between somatic gene mutations and germline gene mutations. For somatic mutated genes, only lung cancer driver genes were plotted. *P*‐values were estimated based on the Poisson‐Binomial distribution and provided by the rediscover package. (B) Co‐occurrent and mutually exclusive association between somatic gene mutations and germline pathway mutations. For somatic mutated genes, only lung cancer driver genes were plotted. *P*‐values were estimated based on the Poisson‐Binomial distribution and provided by the rediscover package. *P*‐values were not adjusted. (C) Distribution of *EGFR* somatic mutations along the CDS of *EGFR* in the patients with and without *TP53* germline P/LP variants (Kolmogorov–Smirnov test). (D) Distribution of *TP53* somatic mutations along the CDS of *TP53* in the patients with and without HR germline P/LP variants (Kolmogorov–Smirnov test). (E–J) Comparison of the contributions of various signatures in the pooled samples with and without germline variants in certain pathways (Chi‐square test). gHR(+) represents presence of germline variants in the homologous recombination repair pathway. Damage Sensor, Damage Sensor pathway; other_DDR, other DNA damage repair pathways; PI3K, PI3K pathway; RTK‐RAS, RTK‐RAS pathway; TGF_Beta, TGF‐Beta pathway; TP53, TP53 pathway.

We firstly analyzed the co‐occurrence and mutually exclusive relationship between germline and somatic variants. Due to the relatively limited germline mutation events, all pairwise comparisons had > 0.05 FDR after multiple testing adjustment. Figure 4A displays germline and somatic gene pairs with < 0.05 *P* value and Fig. 4B displayed germline pathway and somatic gene pairs with < 0.05 *P* value (for somatic genes, only lung cancer driver genes were plotted). Mutually exclusive events between germline and somatic mutations were rare. In fact, at gene level, only *TP53* germline mutations and its somatic mutations showed mutually exclusive association (Fig. 4A), and at pathway level, only germline mutations of the NER pathway were mutually exclusive with somatic *FAT1* mutations (Fig. 4B). Majority associations between germline and somatic mutations were co‐occurrence suggesting that certain genes may be susceptible to P/LP germline variants. For example, somatic mutations in CTCF and EZH2 gene were co‐occurrent with germline mutations in multiple DNA repair associated genes such as *ERCC5*, *POLH*, *XPC*, *CHEK2*, *ERCC3*, *MUTYH*, *XPA*, and *POLE* (Fig. 4A), suggesting the two genes may be susceptible to DNA damage repair deficiency. In addition, somatic mutations in *ARID1A*, *CDC73*, and *SMARCA4* gene were co‐occurrent with germline mutations of the MMR pathway (Fig. 4B); previous studies have found that mutations in *ARID1A* [53], *CDC73* [54], and *SMARCA4* [55] are enriched in microsatellite instable tumors, which supports our findings and demonstrates these genes may be the targets of MMR deficiency. Germline variants can also affect the distribution of somatic mutation loci. For example, the distribution of the mutations of *EGFR* gene along its coding sequence (CDS) in the patients with and without germline *TP53* mutations was significantly different shown by Kolmogorov–Smirnov test (Fig. 4C), so was the distribution of the mutations of *TP53* gene in the patients with and without germline HR pathway mutations (Fig. 4D). Finally, germline variants may impact on the mutation context. Germline mutations in different pathways were associated with distinct somatic mutational signatures. As expected, the contribution of DNA repair deficiency related signatures was generally higher in the samples with germline mutations of the pathways involved in DNA repair, in comparison with those without the germline mutations of the same pathways; on the contrary, the contribution of the signatures derived from other specific etiological factors for example smoking was relatively lower in the samples with germline mutations of the DNA repair pathways (Fig. 4E–H). Interestingly, samples with germline mutations in the RTK‐RAS pathway had a higher contribution of APOBEC signature (Fig. 4I), and samples with germline mutations in the TP53 pathway had a higher contribution of age related signature (Fig. 4J); both were associated with a distinct somatic mutation context pattern compared with the DNA repair related pathways.

## 4 Discussion

In this study, we used a large‐scale cohort to explore the prevalence and landscape of germline P/LP variants in Chinese lung cancer patients. The overall prevalence of germline P/LP variants in our cohort was 9.7%, which was higher than 5–6% of the prevalence previously reported in Chinese lung cancer patients [20, 21] but comparable to 11.8% in the Asia subgroup in a recent large‐sample cohort [56] as well as 14.7% in another large cohort having European individuals as major study subjects [57]. The inconsistency in prevalence may be attributed to patient selection, study design and particularly the tested cancer susceptible genes; nonetheless, our and other large‐scale studies suggest that germline P/LP variants in lung cancer is not as low as thought before. In line with previous studies, pathways involved in DNA damage repair were most susceptible to germline P/LP mutation; the top seven most frequently mutated pathways were all associated with DNA damage repair including HR (2.09%), other DDR (1.28%), TP53 pathway (0.83%), NER (0.70%), FA pathway (0.6%), DNA damage sensor (0.53%), and MMR (0.39%). This also means 2.09% patients harboring HR variants and 0.53% patients harboring DNA damage sensor variants may benefit from PARP inhibitors and ATR inhibitors.

As to most frequently mutated genes, majority are also reported in other studies including *MUTYH*, *TP53*, *BLM*, *BRCA2*, *PALB2*, *WRN*, *BRIP1*, *BRAC1*, *FANCA*, and *FANCD2*. [20, 58]. Unexpectedly, two genes which are seldom reported to link to lung cancer risk, *SBDS* and *TSHR*, had the highest prevalence in our cohort. Germline P/LP variants of *SBDS*, a pathogenic gene of Shwachman–Diamond Syndrome (SDS), have been found to be associated with an increased risk of myeloid malignancy [59, 60], and a recent study reported that a few of patients with malignant pleural mesothelioma carried germline truncation mutation of *SBDS* (p.K62*) [61]. p.K62* truncation mutation is one of the two common variants of *SBDS* gene (another common variant is c.258+2T>C, a splice site variant). Both variants had relatively high frequency among the control population including gnomAD East Asian population [60]. Despite that, SBDS p.K62* variant had a high odds ratio (4.8) in our case–control analysis with gnomAD East Asian dataset as the control. It seems that the P/LP germline variants of *SBDS* not only increase the risk of myeloid malignancy, but also may increase the risk of solid tumors like lung cancer; however, the conclusion needs other studies to validate and the underlying mechanisms also need experimental studies to illustrate. *TSHR* is another gene with a high prevalence of germline P/LP variants. A study showed that TSHR p.F525S was the most prevalent variant in a Chinese congenital hypothyroidism [62]. This variant also had a relatively high odds ratio in our case–control analysis. The association between germline variants of *TSHR* and cancer risk is unclear although a few of studies suggest that certain *TSHR* polymorphism may increase the risk of thyroid cancer [63]. However, thyroid hormones have important functions in cellular proliferation and differentiation, thus, are closely related to the cancer development [64, 65]. Both hyperthyroidism and hypothyroidism are linked to the increase of site‐specific cancer risk [66, 67, 68, 69]. There is evidence to show that hyperthyroidism is associated with an increased risk of lung cancer [66, 70]. Therefore, germline P/LP variants of *TSHR* gene may indirectly affect the cancer risk by potentially altering the level of thyroid hormones in the body. To sum up, the two genes may not be lung cancer specific susceptible genes, and the association of their P/LP variants and cancer risk warrants further investigation. In terms of the other two significant variants in the case–control analysis (FDR < 0.05), germline BRIP p.L340F variant was detected in breast cancer patients [71] and MUTYH p.R19* variant was detected in patients with colorectal cancer [72], clear cell renal cell carcinoma [73], and breast cancer [74].

We recognize there are some limitations in the study. First, because our analysis was confined to the known cancer susceptibility genes, consequently, the pool of candidate genes was constrained. Our study may miss other potential susceptibility loci, genes and pathways. Secondly, our study was a real‐world retrospective study, the phenotype information including clinicopathological features and survival data of the patients was insufficient; therefore, we could not investigate the association of the germline P/LP variants with more phenotypes such as survival.

## 5 Conclusions

In conclusion, our study shows that Chinese lung cancer patients have a relatively high prevalence of germline P/LP variants. The P/LP variants are distributed in multiple pathways and dominated by DNA damage repair‐associated pathways. In addition to the genes such as *TP53*, *ATM*, *BRAC2* etc, by using of the large‐scale cohort, we also found some relatively rare variants which may be associated with a higher risk of lung cancer. However, the association between identified P/LP variants and lung cancer risk needs further studies to verify.

## Conflict of interest

Xiaoying Wu, Xiaojun Fan, Jiaohui Pang, Hua Bao, Jiani Yin, Xue Wu and Yang Shao are employees of Nanjing Geneseeq Technology, Inc. All other authors declared no conflicts of interest.

## Author contributions

ZCL and FL conceived the project and designed the study. ZY and ZRZ analyzed the data and wrote the manuscript. JL, XYW, and XJF carried out the bioinformatics analysis and prepared the figures. JHP, HB, JNY, XW, and YS coordinated the project, collected and curated the data. All authors read and approved the final manuscript.

## Supporting information


**Table S1.** Cancer susceptibility gene list.

**Table S2.** Mutation frequencies of the 87 cancer susceptibility genes with detectable P/LP germline mutations.

## Data Availability

The datasets used and/or analyzed during the current study available from the corresponding author on reasonable request.
